# Classification of Health-Related Social Media Posts: Evaluation of Post Content–Classifier Models and Analysis of User Demographics

**DOI:** 10.2196/14952

**Published:** 2020-04-01

**Authors:** Ryan Rivas, Shouq A Sadah, Yuhang Guo, Vagelis Hristidis

**Affiliations:** 1 Department of Computer Science and Engineering University of California, Riverside Riverside, CA United States

**Keywords:** social media, demographics, classification

## Abstract

**Background:**

The increasing volume of health-related social media activity, where users connect, collaborate, and engage, has increased the significance of analyzing how people use health-related social media.

**Objective:**

The aim of this study was to classify the content (eg, posts that share experiences and seek support) of users who write health-related social media posts and study the effect of user demographics on post content.

**Methods:**

We analyzed two different types of health-related social media: (1) health-related online forums—WebMD and DailyStrength—and (2) general online social networks—Twitter and Google+. We identified several categories of post content and built classifiers to automatically detect these categories. These classifiers were used to study the distribution of categories for various demographic groups.

**Results:**

We achieved an accuracy of at least 84% and a balanced accuracy of at least 0.81 for half of the post content categories in our experiments. In addition, 70.04% (4741/6769) of posts by male WebMD users asked for advice, and male users’ WebMD posts were more likely to ask for medical advice than female users’ posts. The majority of posts on DailyStrength shared experiences, regardless of the gender, age group, or location of their authors. Furthermore, health-related posts on Twitter and Google+ were used to share experiences less frequently than posts on WebMD and DailyStrength.

**Conclusions:**

We studied and analyzed the content of health-related social media posts. Our results can guide health advocates and researchers to better target patient populations based on the application type. Given a research question or an outreach goal, our results can be used to choose the best online forums to answer the question or disseminate a message.

## Introduction

### Background

There is a huge amount of knowledge waiting to be extracted in health-related online social networks and forums, which we collectively refer to as social media. Health-related social media store the interactions of users who are interested in health-related topics [1]. These users share their experiences, share information of friends and family, or seek help for a wide range of health issues [1]. In the United States, more than 60 million Americans have read or collaborated in *health 2.0* resources [2]. In addition, 40% of Americans have doubted a professional opinion when it conflicted with the opinions expressed in health-related social media [2]. Health-related social media widen access to health information for the public, regardless of individuals’ race, age, locality, or education [1].

In this study, we evaluated the content of posts in various health-related social media. We analyzed two types of health-related social media: (1) health-related online forums: WebMD and DailyStrength and (2) general social networks: Google+ and Twitter. This was a 4-step process comprising data collection, identifying post content categories, performing classification experiments, and performing a demographics analysis. We first collected large datasets of posts from each source and identified several categories. Afterward, we identified meaningful categories from randomly selected posts from each source. In our classification experiments, we labeled data from each source and trained classifiers to identify post content categories. Finally, we used classifiers trained on our labeled data to identify categories in the remaining data and analyzed how often posts in these categories are made by various demographic groups.

The goal of this study was to provide researchers with information and tools to support further research. For example, researchers looking for clinical trial participants can use DailyStrength, where users often share experiences about a particular condition, and health advocates seeking to spread awareness about a condition that affects men can use WebMD, where men often ask for advice. To this end, we also made comparisons between platforms to suggest where such a researcher might begin looking. The classifier models built in this study can assist with this task as well as other analyses involving health-related online postings.

### Related Work

#### Analysis of Health-Related Social Media

Many studies have been performed to characterize health-related social media communities. Hackworth and Kunz [3] reported that 80% of Americans have searched the internet for health-related information, more than 60 million Americans are consumers of social networks in the Web 2.0 environment (health 2.0), and consumers, especially those with chronic conditions, are leading the health 2.0 movement by seeking clinical knowledge and emotional support. Wiley et al [4] studied the impact of different characteristics of various social media forums on drug-related content and demonstrated that the characteristics of a social media platform affect several aspects of discussion. Eichstaedt et al [5] predicted the county-level heart disease mortality by capturing the psychological characteristics of local communities through expressed text in Twitter. However, these studies do not describe or compare specific demographics in terms of their post content.

Further work has focused on categorizing health-related posts based on their content. Yu et al [6] performed a preliminary content analysis of D/deaf and hard of hearing discussion forum, AllDeaf, to observe different types of social support behaviors and identify social support features for a future text classification task. Reavley and Pilkington [7] analyzed the content of tweets related to depression and schizophrenia, finding that tweets about depression mostly discussed consumer resources and advertisements, whereas tweets about schizophrenia mostly raised awareness and reported research findings. Lee et al [8] analyzed the content of tweets from health-related Twitter users, finding that they tweet about testable claims and personal experiences. Lopes and Da Silva [9] collected posts from a health-related online forum, MedHelp, and used them to propose and refine a scheme for manually classifying health-related forum posts into 4 categories and a total of 23 subcategories. Our work was built upon these studies by defining our own categories of post content, some of which have analogues in these studies.

#### Health-Related Demographic Analysis

Other work has compared health issues between demographics or examined the demographics within a population participating in health-related research. Krueger et al [10] studied the mortality attributable to a low education level in the United States across several demographics, where they found people with an education level below a high school degree to have a higher mortality rate. Anderson-Bill et al [11] examined the demographics and behavioral and psychosocial characteristics of *Web-health users* (adults who use the Web to find information on health behavior and behavior change) recruited for a Web-based nutrition, physical activity, and weight gain prevention intervention. Their results suggest that users participating in online health interventions are likely “middle-aged, well-educated, upper middle-class women whose detrimental health behaviors put them at risk of obesity, heart disease, some cancers, and diabetes” [11]. These studies describe the demographics of the populations in their studies but do not describe the demographics of health-related social media users.

Previous work has focused on characterizing demographics on health-related social media. Sadah et al [12] analyzed the demographics of health-related social media and found that users of drug review websites and health-related online forums are predominantly women, health-related social media users are generally older than general social media users, black users are underrepresented in health-related social media, users in areas with better access to health care participate more in health-related social media, and the writing level of health-related social media users is lower than the reading level of the general population. Sadah et al [13] also performed a demographic-based content analysis of health-related social media posts to extract top distinctive terms, top drugs and disorders, sentiment, and emotion, finding that the most popular topic varied by demographic, for example, pregnancy was popular with female users, whereas cardiac problems, HIV, and back pain were the most discussed topics by male users. They also found that users with a higher writing level were less likely to express anger in their posts. We expanded upon this work by characterizing and comparing the demographics of health-related social media websites in terms of the frequency of post content categories.

### Text Classification in Social Media

Text classification is frequently employed by researchers to gain insights into social media users and trends, both in and out of health-related settings. Sadilek et al [14] studied the spread of infectious diseases by analyzing Twitter data using a support vector machine (SVM) model. Huh et al [15] developed a naïve Bayes model to help WebMD moderators find posts they would likely respond to. Nikfarjam et al [16] proposed a machine learning–based tagger to extract adverse drug reactions from health-related social media. Mislove et al [17] estimated the gender and ethnicity of Twitter users using the reported first name and last name. Sadah et al [12] expanded upon the work of Mislove et al [17] by considering screen names in estimating gender. In this study, we used text classification techniques to identify categories of post content in health-related social media and used the techniques proposed in the studies by Sadah et al [12] and Mislove et al [17] to study the frequency of these categories within several demographics.

## Methods

### Datasets

For health-related online forums, we selected 2 different websites, WebMD and DailyStrength. The reason for selecting 2 health-related online forums is to cover the different types of health-related online forums that they each represent. Although WebMD consists of multiple health communities where people ask questions and get responses from the community members [18], DailyStrength enables patients to exchange experiences and treatments, discuss daily struggles and successes, and receive emotional support [19]. For each post collected from these websites, we extracted the URL, title, author’s username, post time, the body of the post, and the name of the message board. For each user of a collected post, we also collected the author’s age, friends, gender, and location, where applicable. As crawling of these sites has been performed at different times, some of the data we have collected do not reflect the current availability of certain attributes because of website format changes, for example, age and gender are currently available from WebMD user profiles but were not available before. In this study, the selection of demographic attributes we used for a source is based on the availability reflected by the majority of posts collected from that source, for example, most of the WebMD posts in our data were collected before age and gender were available, thus we did not use these attributes for an analysis of WebMD user demographics. We restricted the posts used from these sources to the first post in each thread. In our analysis, we used the post body, post title, message board name, and username from WebMD and the post body, post title, message board name, and user’s gender, age, and location from DailyStrength.

For general social networks, we chose Twitter and Google+ as they offer interfaces to easily collect their data (in contrast to Facebook). For each Twitter post, we collected the post content, post time, location, and the author’s username and location. For each Google+ post we collected the title, post time, update time, the post content, the location, and the author’s username, first and last names, age, gender, and location. As Twitter and Google+ are general social networks, we used 274 representative health-related keywords to filter them as follows: (1) Drugs: from the most prescriptions dispensed from RxList [20], we selected the 200 most popular drugs. By removing the variants of the same drug (eg, different milligram dosages), the final list of drugs contained 124 unique drug names. (2) Hashtags: 11 popular health-related Twitter hashtags, such as #BCSM (Breast Cancer and Social Media). (3) Disorders: 81 frequently discussed disorders, such as AIDS and asthma. (4) Pharmaceuticals: the names of the 12 largest pharmaceutical companies, such as Novartis. (5) Insurance: the names of the 44 biggest insurance companies, such as Aetna and Shield. (6) General health-related keywords “healthcare” and “health insurance.” To reach the final keyword counts for hashtags, disorders, pharmaceuticals, and insurance, we sampled each keyword from a larger list for each of these categories and kept keywords with a high ratio of health-related posts. In our analysis, we used the tweet body, user’s first and last name, and user’s location from Twitter and post body, post title, and user’s gender, age, first and last name, and location from Google+.

To filter Twitter with the health-related keyword list to retrieve relevant tweets for TwitterHealth, we used the Twitter streaming application programming interface (API) [21]. Similarly, we used Google+ API [22] to extract the relevant posts for Google+Health. For health-related online forums WebMD and DailyStrength, we built a crawler for each website in Java using jsoup [23], a library to extract and parse HTML content. Table 1 lists for each source the number of posts collected, the date ranges of collected posts, and whether the demographic attributes used in this study are present, and Table 2 lists the distribution of demographics for each source across each demographic attribute. For all 4 of these sources, we did not specifically focus our search on English-language posts aside from using English drug names; however, the majority of posts collected from these sources were in the English language.

**Table 1:** List of all sources used with their number of posts, date range of posts, and the available demographic attributes.

Source	Number of posts	Date range	Gender	Age	Ethnicity	Location
TwitterHealth [24]	11,637,888	May 2, 2013 to November 11, 2013	Gender classifier [17]	No^a^	Ethnicity classifier [17]	Yes^b^
Google+Health [25]	186,666	August 24, 2009 to January 5, 2014	Yes	Yes	Ethnicity classifier [17]	Yes
DailyStrength [26]	1,319,622	June 21, 2006 to December 3, 2017	Yes	Yes	No	Yes
WebMD [27]	318,297	December 24, 2006 to May 11, 2019	Gender classifier [12]	No	No	No

^a^The demographic attribute is not provided by the source and no classifier is used because of low accuracy.

^b^The demographic attribute is provided by the source.

**Table 2:** Demographics of users from each source.

Attribute and demographic		TwitterHealth, %	Google+Health, %	DailyStrength, n (%)	WebMD, n (%)
**Gender**					
	Male	48.19^a^	64.64^a^	95,269 (17.26)^b^	6769 (32.41)^b^
	Female	51.81^a^	35.36^a^	456,600 (82.74)^b^	14,117 (67.59)^b^
**Age (years)**					
	0-17	N/A^c^	3.42^a^	6656 (1.33)^b^	N/A
	18-34	N/A	53.21^a^	187,966 (37.55)^b^	N/A
	35-44	N/A	21.89^a^	126,646 (25.30)^b^	N/A
	45-64	N/A	19.02^a^	149,487 (29.86)^b^	N/A
	≥65	N/A	2.46^a^	29,847 (5.96)^b^	N/A
**Ethnicity**					
	Asian	3.24^a^	5.60^a^	N/A	N/A
	Black	0.30^a^	0.30^a^	N/A	N/A
	Hispanic	23.50^a^	17.40^a^	N/A	N/A
	White	73.00^a^	76.60^a^	N/A	N/A
**Region**					
	Northeast	165,531 (19.83)^d^	2598 (17.86)^d^	73,221 (19.58)^b^	N/A
	Midwest	174,620 (20.92)^d^	2393 (16.45)^d^	84,302 (22.55)^b^	N/A
	South	313,350 (37.53)^d^	4863 (33.44)^d^	123,556 (33.05)^b^	N/A
	West	181,400 (21.73)^d^	4690 (32.25)^d^	92,809 (24.82)^b^	N/A

^a^Based on Sadah et al [12].

^b^Calculated with user data collected or estimated from this study.

^c^N/A: not applicable.

^d^Calculated from user counts reported in the study by Sadah et al [13].

### Identifying Post Contents

From each source, we randomly selected 500 posts. We then manually identified the different categories of shared content for each type of health-related social media. As shown in Table 3, we identified 9 different categories. The first 4 categories were identified for both types of health-related social media (hence, all 4 sources). Of these first 4 categories, 3 were also identified by Lopes and Da Silva [9], for example, *share experiences*, which we defined as posts in which a user shared a personal experience related to a health-related topic. This is similar to their *sharing personal experiences* category, except that we did not restrict our definition to experiences shared in response to another post. *About family* has no equivalent in their scheme, but it can be covered by other categories that they have defined, for example, by asking a specific question about or expressing sadness over a family member’s illness. Our share experiences category was also similar to categories in other work, for example, the *personal experience of mental illness* category in the study by Reavley and Pilkington [7], the *personal* category from Lee et al [8], the *personal event* category from Robillard et al [28], and the *first-hand experience* category from Alvaro et al [29]. As Twitter and Google+ are more news-based social media, we identified 5 additional categories from these sources. *Educational material* can be considered equivalent to the *teaching* category defined by Lopes and Da Silva [9]. Despite the differences between the categories we defined and those proposed by Lopes and Da Silva [9], we believed that our categories are sufficient for a *proof of concept* for automatic post content category classification in the two types of health-related social media that we investigated. It should be noted that the identification of specific experiences is outside the scope of this study; the *share experiences* category is a catch-all for any experiences shared in a health-related post from any source.

We asked 3 graduate students to label the selected data from WebMD, Twitter, and Google+; we used a majority vote as the final result for each of these sources. Table 4 lists the intercoder agreement as given by a Krippendorff alpha for our labeled datasets from WebMD, Twitter, and Google+. The selected DailyStrength data were labeled by the labeler with the highest agreement with the majority averaged over each category from the other 3 sources (average alpha=.680). As shown in Table 5, the distribution of categories in each source is different, for example, the share experiences category is more common in health-related online forums (WebMD and DailyStrength).

**Table 3:** List of all identified categories for health-related online forums and general social networks.

Category	Health-related online forums	General social networks	Example
Share experiences	Yes	Yes	“I could not work after Tylenol.” “I have taken Lipitor every day.”
Ask for specific medical advice or information	Yes	Yes	“Is honey allowed for diabetics?”
Request or give psychological support	Yes	Yes	“I hope your diabetes is under control.” “We’re thinking of you.”
About family (not about self)	Yes	Yes	“My son is now nine months old and teething like crazy.”
Share news	No	Yes	“Kaiser Permanente Invites Software Developers To Build Apps—Forbes. http://feedly.com/k/Zojwq”
Jokes	No	Yes	“Got any jokes about Sodium Hypobromite? NaBro.”
Advertisements	No	Yes	“Check out these two vitamins for one recipe! http://bit.ly/1471dbn”
Personal opinion	No	Yes	“Main frustration of lupus is losing the ability to do things that used to be normal”
Educational material	No	Yes	“Side Effects of Alzheimer’s and Dementia Drugs http://bit.ly/cK7L1f”

**Table 4:** Intercoder agreement for our labeled datasets (Krippendorff alpha).

Category	WebMD	TwitterHealth	Google+Health
Share experiences	0.349	0.446	0.109
Ask for specific medical advice or information	0.768	0.225	0.108
Request or give psychological support	0.219	0.090	−0.007
About family (not about self)	0.736	0.322	−0.010
Share news	N/A^a^	0.083	0.083
Jokes	N/A	0.177	0.029
Advertisement	N/A	0.220	0.107
Personal opinion	N/A	0.103	0.038
Educational material	N/A	0.164	0.091

^a^N/A: not applicable.

**Table 5:** Percentages of categories in each source from the labeled data (N=500).

Category	WebMD, n (%)	DailyStrength, n (%)	TwitterHealth, n (%)	Google+Health, n (%)
Share experiences	236 (47.2)	400 (80.0)	74 (14.8)	65 (13.0)
Ask for specific medical advice or information	270 (54.0)	173 (34.6)	3 (0.6)	10 (2.0)
Request or give psychological support	126 (25.2)	247 (49.4)	9 (1.8)	7 (1.4)
About family (not about self)	68 (13.6)	37 (7.4)	5 (1.0)	34 (6.8)
Share news	N/A^a^	N/A	56 (11.2)	145 (28.9)
Jokes	N/A	N/A	38 (7.6)	33 (6.6)
Advertisement	N/A	N/A	26 (5.2)	70 (14.0)
Personal opinion	N/A	N/A	35 (7.0)	84 (16.8)
Educational material	N/A	N/A	36 (7.2)	137 (25.7)

^a^N/A: not applicable.

### Bot Filtering

We examined the impact of automated accounts (ie, *bots*) on our study using OSoMe’s Botometer (formerly BotOrNot, Indiana University) [30], a tool that estimates how likely a Twitter account is to be a bot. We used the Botometer API to score each account that has a tweet in our initial sample of 500. The API assigned each of the 345 accounts that were still active a score in the range 0 to 1, with higher scores corresponding to a higher likelihood of an automated account. We manually evaluated each account with a score above 0.5. With this threshold, which was chosen because it is a natural choice that avoids possible bias from a more arbitrary choice of threshold value, we found a total of 33 likely bot accounts. We found that tweets from these accounts make up a substantial portion of the categories share news (11 tweets), advertisement (12 tweets), and educational material (10 tweets). As Botometer’s API rate limit makes removing all bot tweets from our Twitter corpus of over 11 million tweets unfeasible, we instead randomly selected 1000 posts from each day in the date range of our Twitter data. For each author of these selected posts, we again used Botometer to evaluate the likelihood of an automated account, removing tweets from accounts with a score above 0.5 for a total of 142,411 tweets used in our analysis.

We also manually examined 100 posts each from WebMD and DailyStrength to determine the prevalence of bots on these websites, which consisted of one of the authors reading each of these posts and determining whether or not it appeared to be posted by a spambot. In the context of online forums, a spambot is an automated agent that posts promotional content [31]. By this criterion, none of the posts examined appeared to have been posted by a bot. Although this does not guarantee that there are no posts from bots in the data from these websites used in our study, it does suggest that posts from bots may be much less prevalent in these sources, likely because of the smaller volume of posts and more active moderation compared with Twitter and Google+.

### Building Post Content Classifiers

For each category, we performed binary classification experiments with three classifier algorithms: random forest [32], linear SVM [33], and convolutional neural network (CNN) [34]. We first extracted and concatenated the features shown in Table 6. These features include the title of a post, the main text of a post (body), and the name of the message board that contains the post (board name). For the random forest and SVM classifiers, we converted the features to a term frequency-inverse document frequency vector with stop words removed and the remaining words lemmatized. For the CNN classifier, we converted the features to sets of fastText [35] vectors pretrained on Wikipedia. For all classifiers, we applied class weights to the training data such that the weight of the positive class (the post is in the category) is balanced with the weight of the negative class (the post is not in the category). These weights are used with random forest and SVM according to their implementations by Pedregosa et al [36], whereas CNN uses oversampling of the least frequent class as recommended by Buda et al [37].

To build the classifiers, we excluded the categories where the percentage is less than 10.0% (50/500), and for the rest, we first split the labeled data to two datasets as follows: (1) a training dataset (450 posts) and (2) a test dataset (50 posts), held out for a final test after training is complete. Afterward, for each classifier algorithm, we trained each classifier by varying the hyperparameters shown in Table 7, considering each combination of hyperparameter values. For all combinations, we performed a 5-fold cross-validation on the training dataset to select the combination of hyperparameter values with the highest balanced accuracy [38]. Finally, we used these hyperparameter values to create a model trained on the full training dataset and tested this model on the test dataset that was held out before the cross-validation experiments. Note that we did not use a nested cross-validation, as our goal in these experiments was to find a single combination of hyperparameter values that we could use to apply a sufficiently accurate classifier model to the rest of our data.

Table 8 shows the classifiers’ accuracy for WebMD, DailyStrength, Twitter, and Google+. We have shown only the classifiers for categories that have more than 10% of labeled data.

For the remainder of our analysis, we only considered source-category combinations with a classifier that achieved a balanced accuracy higher than 0.75.

For the source-category combinations that did not have a classifier that achieved a balanced accuracy of at least 0.75, we performed another round of experiments in which we attempted to classify posts using the best-performing classifier trained on a corresponding category from another source, for example, random forest for share experiences from WebMD. In these experiments, we used 500 posts from one source for training and 500 posts from another source for testing and again finding the best combination of hyperparameters via a 5-fold cross-validation of the training data. Table 9 shows the results of these experiments. Classifiers trained on the DailyStrength and Twitter data achieved a balanced accuracy of over 0.75 on the share experiences category from Google+, so we added this category to the set of categories considered for further analysis. For each category in this set, we used the model with the highest balanced accuracy for that category to label the rest of the data. We reported our findings on the frequency of these categories by several demographics according to their respective classifiers in the Results section.

**Table 6:** All classifiers’ training features.

Source	Extracted features
WebMD	Title, body, and board name
DailyStrength	Title, body, and board name
Google+	Title and body
Twitter	Body

**Table 7:** Classifier hyperparameter values evaluated in our experiments.

Classifier and hyperparameter		Values
**Random forest**		
	Maximum tree depth	2, 4, 8, 16, 32, 64
	Number of trees, n	10, 100, 1000
**Support vector machine**		
	C	0.001, 0.01, 0.1, 1, 10
	Loss function	Hinge, squared hinge
**Convolutional neural network**		
	Filter window sizes	(2, 3, 4), (3, 4, 5), (4, 5, 6)
	Feature maps per filter window size, n	100, 200, 300, 400, 500, 600

**Table 8:** Classifier results for each category (N=50).

Source and category		Random forest		Support vector machine		Convolutional neural network	
		Accuracy, n (%)	Balanced accuracy	Accuracy, n (%)	Balanced accuracy	Accuracy, n (%)	Balanced accuracy
**WebMD**							
	Share experiences^a^	41 (82)	0.83^b^	41 (82)	0.81	41 (82)	0.82
	Ask for specific medical advice or information^a^	40 (80)	0.82	41 (82)	0.83^b^	37 (74)	0.76
	Request or give psychological support^a^	39 (78)	0.71	43 (86)	0.8 ^b^	38 (76)	0.68
	About Family (Not about self)^a^	38 (76)	0.56	40 (80)	0.89^b^	47 (94)	0.81
**DailyStrength**							
	Share experiences^a^	41 (82)	0.80	40 (80)	0.70	41 (82)	0.82^b^
	Ask for specific medical advice or information^a^	39 (78)	0.71	38 (76)	0.70	37 (74)	0.7 ^b^
	Request or give psychological support	34 (68)	0.68	33 (66)	0.65	38 (76)	0.68^b^
**TwitterHealth**							
	Share experiences^a^	39 (78)	0.77	41 (82)	0.82^b^	43 (86)	0.74
	Share news^a^	41 (82)	0.64	40 (80)	0.73	47 (94)	0.81
**Google+Health**							
	Share experiences	44 (88)	0.48	35 (70)	0.72^b^	45 (90)	0.60
	Share news	26 (52)	0.48	28 (56)	0.52	33 (66)	0.59^b^
	Advertisement	38 (76)	0.59	24 (48)	0.53	42 (84)	0.6 ^b^
	Personal opinion	39 (78)	0.48	37 (74)	0.71^b^	42 (84)	0.60
	Educational material^a^	40 (80)	0.66	34 (68)	0.76	41 (82)	0.79^b^

^a^The category of each source-category combination with at least one classifier that achieved a balanced accuracy of at least 0.75.

^b^The highest balanced accuracy for each source-category combination.

**Table 9:** Results of classifiers trained on a corresponding category from another source (N=500).

Training source	Test source	Category	Classifier	Accuracy, n (%)	Balanced accuracy
WebMD	DailyStrength	Psychological support	SVM^a^	328 (65.6)	0.656
WebMD	Google+Health	Share experiences	Random forest	428 (85.6)	0.584
DailyStrength	*Google+Health* ^b^	*Share experiences*	CNN^c^	383 (76.6)	*0.800*
Twitter	*Google+Health*	*Share experiences*	SVM	408 (81.6)	*0.770*
Twitter	Google+Health	Share news	CNN	360 (72.0)	0.562

^a^SVM: support vector machine.

^b^The test source, category, and balanced accuracy of each classifier that achieved a balanced accuracy of at least 0.75 are italicized for emphasis.

^c^CNN: convolutional neural network.

### Demographic Analysis

We chose four demographic attributes as shown in Table 1: gender, age, ethnicity, and location. Where possible, we extracted these attributes from user profiles. These attributes are not available for every source, so we used existing classifier models where available to estimate their values. Specifically, we used the classifiers from Mislove et al [17] to estimate gender for Twitter users and ethnicity for both Twitter and Google+ users. To estimate gender for WebMD users, we used the classifier from Sadah et al [12], an extension of the classifier by Mislove et al that considers a user’s screen name when the user’s first name is not present. These classifiers use the 1000 most popular male and female birth names reported by the US Social Security Administration for each year from 1935 to 1995 as ground truth for gender and the distribution of ethnicities for each last name as reported by the 2000 US Census as ground truth for ethnicity. For each of these attributes, we used the data labeled by our post content category classifiers to determine how frequently users of each demographic write a post with one of these categories, for example, the percentage of posts made by male users in which a user shared his experiences. When comparing these percentages, we calculated statistical significance via a Pearson chi-square test. Note that a post can be in more than one category, for example, a post can both share experiences and ask for medical advice.

### Top Distinctive Message Boards

For each combination of demographic and category (eg, male and share experiences) analyzed in WebMD and DailyStrength, we found the most distinctive message boards for that combination. For WebMD, we considered only boards that have at least 0.01% of posts for a given combination, or 30 if 0.01% is less than 30. Owing to the large number of message boards on DailyStrength (1608 analyzed in this study), we reduced this restriction to only consider boards with at least 30 posts for a given combination. We then determined distinctiveness by calculating the relative difference of each board. On the basis of the calculation for top distinctive terms by Sadah et al [13], we calculated the relative difference of board *b* within the combination of category *c* and demographic *b* of demographic attribute *a* as shown in equation (1):

RelDifcd(b)=[Freqcd(b)−AvgFreqca(b)]/AvgFreqca(b) (1),

where *Freq_cd_(b)* is the normalized frequency of posts on board *b* in category *c* by a user in demographic *d*, for example, the number of posts on the WebMD Breast Cancer message board that share experiences and were written by a female user divided by the number of posts on WebMD that share experiences and were written by a female user. *AvgFreq_ca_(b)* is the average *Freq_cd_(b)* across all demographics *d* within the demographic attribute *a*, for example, male and female for the demographic attribute, gender.

## Results

### Demographics

In this section, we presented the categories’ results by each demographic where possible. For age demographics, we organized users into five groups: 0 to 17 years, 18 to 34 years, 35 to 44 years, 46 to 64 years, and older than 65 years. For ethnicity, we considered four possibilities: Asian, black, Hispanic, and white. For location, we considered the four regions designated by the US Census Bureau: Midwest, Northeast, South, and West. As explained in the Methods section, we considered the following categories for each source: (1) WebMD: share experiences, ask for advice, psychological support, and about family; (2) DailyStrength: share experiences and ask for advice; (3) TwitterHealth: share experiences and share news; and (4) Google+Health: share experiences and educational material.

### WebMD

As shown in Table 1, our WebMD dataset includes gender predicted by the gender classifier from Sadah et al [12]. Therefore, we have reported the distribution of gender among its categories. Table 10 shows the frequency of posts made by male and female users for each category. We found that 70.04% (4741/6769) of posts written by male WebMD users asked for advice, compared with 45.14% (6372/14,117) of posts by female users (*P*<.001). Table 11 shows the top 10 most distinctive WebMD message boards by the number of posts for each combination of gender and category. Unsurprisingly, these results show that female users were more likely to post on boards about pregnancy and parenting than males in all categories, whereas male users were more likely to discuss men’s health issues. Men also gave psychological support and discussed family members on the message board for the infertility drug, Clomid, more frequently than women.

**Table 10:** WebMD category frequency by gender.

Category	Gender, n (%)	
	Male (n=6769)	Female (n=14,117)
Share experiences	3290 (48.60)	4835 (34.25)
Ask for advice	4741 (70.04)	6372 (45.14)
Psychological support	1914 (28.28)	5515 (39.07)
About family	1986 (29.34)	3623 (25.66)

**Table 11:** Top 10 most distinctive WebMD message boards for male and female users in each category.

Gender	Share experiences	Ask for advice	Psychological support	About family
Male	Men’s Health Erectile Dysfunction Relationships and Coping Cholesterol Management Epilepsy Depression Allergies Oral Health Knee & Hip Replacement Ear, Nose & Throat	Erectile Dysfunction Cholesterol Management Men’s Health HIV/AIDS Depression Epilepsy Prostate Cancer Sports Medicine Pain Management Ear, Nose & Throat	Relationships and Coping Epilepsy Depression Back Pain Heart Disease Pain Management Anxiety & Panic Clomid Diabetes Parenting: 4 & 5-Year-Olds	Relationships and Coping Depression Erectile Dysfunction Back Pain Clomid Epilepsy Anxiety & Panic Pain Management Sleep Disorders Digestive Disorders
Female	Sexual Abuse Survivors Support Trying to Conceive: 12 Months, Still Trying Endometriosis Breast Cancer Infertility Treatment Pregnancy: After Infertility Pregnancy: After 35 Parenting: Elementary Ages Self-Harm Menopause	Trying to Conceive: 12 Months, Still Trying Infertility Treatment Dieting Club: 25-50 Lbs Parenting: Preteens & Teenagers Skin & Beauty Breast Cancer Food & Cooking Lupus Parenting: 3-Year-Olds Parenting: 9-12 Months	Chronic Fatigue Syndrome Lupus Sexual Abuse Survivors Support Breast Cancer Endometriosis Dieting Club: 10-25 Lbs Trying to Conceive: 12 Months, Still Trying Pregnancy: After 35 Dieting Club: 100+ Lbs Pregnancy: After Infertility	Sexual Abuse Survivors Support Pregnancy: After 35 Trying to Conceive: 12 Months, Still Trying Trying to Conceive: After Loss Breast Cancer Self-Harm Parenting: Preteens & Teenagers Parenting: 9-12 Months Dieting Club: 50-100 Lbs Parenting: 6-9 Months

### DailyStrength

For our DailyStrength demographic attributes, gender, age, and location, we reported the results for the categories share experiences and ask for advice. Table 12 shows the category frequencies for each demographic. The majority of posts (over 80%) from every demographic share experiences; but among the different age demographics, we saw a clear decline in frequency as age increases, from 92.77% (6175/6656) for users aged younger than 18 years to 81.82% (24,420/29,847) for users 65 years and older (*P*<.001). The frequency of posts that ask for advice is similar for almost every demographic (30%-40%), with the exception of posts from users younger than 18 years 25.45% (1694/6656). *P*<.001 for all comparisons between users younger than 18 years and other age groups.

Tables 13-15 show the top 10 most distinctive DailyStrength message boards by the number of posts for each combination of gender and category, age group and category, and location and category, respectively. From these lists, we saw a wider variety of topics compared with WebMD, likely because of the large number of message boards on DailyStrength. However, we still saw some trends when considering broader topics. Male users tend to share experiences on message boards related to personal and social issues. Both male and female users asked for advice most frequently on boards related to physical conditions.

We also observed a general tendency for younger users (aged younger than 45 years) to share experiences on message boards about personal and social issues, whereas older users favored message boards for general support and discussion. Users in all age groups frequently asked for advice about physical conditions. We found no clear trend in sharing experiences when evaluating census regions, but we saw that users from the Northeast region share experiences about physical and psychological conditions, whereas users from the West region often shared experiences on message boards for general support and discussion. Users from all regions frequently asked for advice about physical conditions except the West, whose users tended to ask for advice on message boards for general support and discussion. Note that there are fewer than 10 message boards listed for users of age 0 to 17 years who asked for advice in Table 14 because of the lack of message boards that also met our restriction of having at least 30 of these posts.

**Table 12:** DailyStrength category frequency by gender, age, and location.

Attribute and demographic		Total number of participants	Share experiences, n (%)	Ask for advice, n (%)
**Gender**				
	Male	95,269	78,760 (82.67)	31,706 (33.28)
	Female	456,600	409,640 (89.72)	167,867 (36.76)
**Age group (years)**				
	0-17	6656	6175 (92.77)	1694 (25.45)
	18-34	187,966	173,226 (92.16)	65,191 (34.68)
	35-44	126,646	113,796 (89.85)	48,335 (38.17)
	45-64	149,487	127,089 (85.02)	54,008 (36.13)
	≥65	29,847	24,420 (81.82)	10,581 (35.45)
**Region**				
	Northeast	73,221	65,761 (89.81)	28,196 (38.51)
	Midwest	123,556	76,630 (90.90)	31,600 (37.48)
	South	123,556	110,597 (89.51)	46,933 (37.99)
	West	92,809	76,797 (82.75)	31,481 (33.92)

**Table 13:** Top 10 most distinctive DailyStrength message boards for male and female users in each category.

Gender	Share experiences	Ask for advice
Male	Vow To Live LGBT Against Suicide Christian Church 24.7 Ministry Gay Men’s Challenges Single Dads GOYA Dealing with Diabetes2 and remembering Goldi A Child Abuse Survivors Group CALM and EASY GAMES Financial Challenges Liars Anonymous	A Laughter Club Dealing with Diabetes2 and remembering Goldi Impotence & Erectile Dysfunction Sex/Pornography Addiction High Cholesterol Tinnitus, Deafness and Ear Problems Urinary Incontinence Atrial Fibrillation (AFib) MRSA LDN .. Low Dose Naltrexone
Female	helping with the housework Lesbian Relationship Challenges prompts AlAnon One Day At A Time Daughters of Abusive Mothers Breastfeeding Parenting Toddlers (1-3) Post-Partum Depression Infertility Vulvar Cancer	Pregnancy Menopause Trying To Conceive Miscarriage Polycystic Ovarian Syndrome (PCOS) Family & Friends of Bipolar WHY WEIGHT? LET’S LOSE WEIGHT AND FEEL GREAT! Infertility Vulvar Cancer Breastfeeding

**Table 14:** Top 10 most distinctive DailyStrength message boards for each age group in each category.

Age group (years)	Share experiences	Ask for advice
0-17	Weight Loss For Teens Gay & Lesbian Teens Depression–Teen Bipolar Disorder–Teen Self-Injury Transgender Depression Coming Out Bisexuality Eating Disorders	Weight Loss For Teens Depression–Teen Self-Injury Eating Disorders Anxiety
18-34	Sunny and Peaceful Skies Parenting Toddlers (1-3) Daily Positive Thoughts Trying To Conceive Parenting Newborns & Infants (0-1) College Stress Arnold-Chiari Malformation ALL MOODY BLUES Career Changes Cerebral Palsy	Trying To Conceive Neuropathy Pregnancy Miscarriage Polycystic Ovarian Syndrome (PCOS) Cerebral Palsy Endometriosis Pseudotumor Cerebri Sexually Transmitted Diseases–Female Schizophrenia
35-44	Vow To Live LGBT Against Suicide Parenting 'Tweens (9-12) Twins, Triplets & More Self-Hate Syndrome Parents Whose children have been sexually abused HOPEFUL HEARTS...LIVING AGAIN AFTER THE LOSS Neurofibromatosis Breastfeeding Hyperparathyroidism Stillbirth	kindredspirits Hyperparathyroidism Multiple Sclerosis (MS) Pseudotumor Cerebri Allergies Hemochromatosis Hypothyroidism Addison’s Disease MCTD Graves’ Disease
45-64	acoa sanctuary prompts Christians with MS InHisCare Bible Study The Serenity Room Ticked off about Lyme Biblical Studies and Archaeology Alanon support group Just support WHY WEIGHT? LET’S LOSE WEIGHT AND FEEL GREAT!	WHY WEIGHT? LETS LOSE WEIGHT AND FEEL GREAT! MS People Dealing with MS Pain Dealing with Diabetes2 and remembering Goldi Multiple Myeloma Menopause High Cholesterol LDN .. Low Dose Naltrexone Myofascial Pain Syndrome Neurocardiogenic Syncope Amputees
≥65	Banana A Little Bit Of Kindness Goes A long Way! AlAnon One Day At A Time VOICES OF RECOVERY The Walking Group The Front Porch Over The Fence Muscular Dystrophies CALM and EASY GAMES movie lovers	AlAnon One Day At A Time VOICES OF RECOVERY I can’t HEAR you! COPD & Emphysema Meniere’s Disease Parkinson’s Disease Sleep Apnea Interstitial Cystitis (IC) Atrial Fibrillation (AFib) Acromegaly

**Table 15:** Top 10 most distinctive DailyStrength message boards for each region in each category.

Region	Share experiences	Ask for advice
Northeast	WHY WEIGHT? LET’S LOSE WEIGHT AND FEEL GREAT! Self-Hate Syndrome Smoking Addiction & Recovery Urinary Incontinence Families of Prisoners Agoraphobia & Social Anxiety Cocaine Addiction & Recovery Obesity CHRISTIAN PARENTS of ESTRANGED ADULT CHILDREN Brain Injury	WHY WEIGHT? LET’S LOSE WEIGHT AND FEEL GREAT! Obesity Hidradenitis Suppurativa Endometriosis Deep Vein Thrombosis (DVT) Atrial Fibrillation (AFib) Diets & Weight Maintenance Gastritis Polycystic Kidney Disease (PKD) Hypothyroidism
Midwest	Just support acoa sanctuary helping with the housework kindredspirits The Coffee Shop aa Spoken Here Highly Sensitive People HSP Financial Challenges I can’t HEAR you! Pseudotumor Cerebri	kindredspirits Neurocardiogenic Syncope Pseudotumor Cerebri Gastritis Irritable Bowel Syndrome (IBS) COPD & Emphysema Parkinson’s Disease Polycystic Kidney Disease (PKD) Pancreatitis Graves’ Disease
South	prompts Beyond Medication InHisCare Bible Study Ticked off about Lyme Muscular Dystrophies aa friends Anxiety and POSITIVE CHOICES Games for Fun and Relaxation MS People Dealing with MS Pain Parents Whose children have been sexually abused	MS People Dealing with MS Pain High Cholesterol Cirrhosis Polymyositis & Dermatomyositis Addison’s Disease Meniere’s Disease MCTD Trying To Conceive Endometriosis Polycystic Ovarian Syndrome (PCOS)
West	A Little Bit Of Kindness Goes A long Way! The Walking Group Alanon support group VOICES OF RECOVERY AlAnon One Day At A Time BIBLICAL STUDIES The Sunflower group My Favorite Things. FrIeNdShIpRoOm three prayerpraise	AlAnon One Day At A Time Banana The Sunflower group WINGS VOICES OF RECOVERY A Laughter Club FrIeNdShIpRoOm Myofascial Pain Syndrome Hemochromatosis Colon Cancer

### Twitter

For our Twitter demographic attributes, gender, ethnicity, and location, with gender and ethnicity predicted by the classifier from Mislove et al [17], we reported the results for categories share experiences and share news using our sample of 142,411 tweets in Table 16. As described in the Methods section, this dataset was created from our full corpus by first sampling 1000 posts for each day represented in the dataset and then pruning tweets from likely bot accounts. All demographics analyzed shared experiences more often than they shared news. Hispanic users had the largest difference, with 29.16% (826/2833) of them shared experiences versus 5.47% (155/2833) of them shared news (*P*<.001). Users from the Northeast census region had the smallest difference, with 20.38% (1093/5362) of them shared experiences versus 10.16% (545/5362) of them shared news; *P*<.001. Where comparison is possible between these demographics and their counterparts in WebMD and DailyStrength, we saw that Twitter users shared experiences less frequently (*P*<.001 for all such comparisons).

**Table 16:** Twitter category frequency by gender, ethnicity, and location.

Attribute and demographic		Total number of participants	Share experiences, n (%)	Share news, n (%)
**Gender**				
	Male	16,092	3188 (19.81)	1277 (7.94)
	Female	17,850	4835 (27.09)	1091 (6.11)
**Ethnicity**				
	Asian	626	166 (26.52)	34 (5.43)
	Black	56	12 (21)	3 (5)
	Hispanic	2833	826 (29.16)	155 (5.47)
	White	9992	2259 (22.61)	728 (7.29)
**Region**				
	Northeast	5362	1093 (20.38)	545 (10.16)
	Midwest	4686	1084 (23.13)	380 (8.11)
	South	9855	2162 (21.94)	850 (8.63)
	West	5448	1164 (21.37)	515 (9.45)

We also performed this analysis on our full Twitter dataset of 11,637,888 tweets. We compared these results with the results shown in Table 16 and found that the differences were generally not statistically significant (with statistical significance defined as *P*<.05) for the share experiences category but were significant for all but one demographic in the share news category. These findings agree with our evaluation of bot likelihood using our initial sample of 500 tweets, where we found that the share news category had a substantial number of tweets from likely bot accounts, but the share experiences category did not. The *P* values of these comparisons are shown in Table 17.

**Table 17:** *P* values of comparisons between Twitter results using pruned data and results using all data.

Category	Male	Female	Asian	Black	Hispanic	White	Northeast	Midwest	South	West
Share Experiences	<.001	.47	.24	.80	.68	.15	.13	.048	.002	<.001
Share News	<.001	<.001	<.001	.23	<.001	<.001	<.001	<.001	<.001	<.001

### Google+

Our Google+ demographic attributes include gender, age, ethnicity, and location, with ethnicity predicted by the classifier from Mislove et al [17], and for these attributes we reported the results from the share experiences and educational material categories in Table 18. As classifiers trained on our labeled Google+ dataset did not achieve a sufficiently high balanced accuracy for the share experiences category, we considered classifiers trained on the labeled DailyStrength and Twitter data as described in the Methods section. The full set of Google+ posts were classified as 34.13% (63,709/186,666) share experiences by the DailyStrength-trained classifier and 18.83% (35,149/186,666) share experiences by the Twitter-trained classifier. As the latter distribution of the share experiences category is closer to the distribution reported in Table 5, 13.0% (65/500), we used the Twitter-trained classifier for the remainder of our analysis in the share experiences category.

From these results, we saw that most demographics appeared to share experiences more frequently than the set of all Google+ users. This is likely the effect of a bias toward users who chose to report these attributes (or a real name, in the case of ethnicity). When comparing how often a demographic shares experiences with how often posts from users with no data on that demographic’s corresponding attribute share experiences (eg, posts from men vs posts from users who did not report gender), we found that *P*<.001 for all such comparisons except for users aged ≥65 years (*P*=.83). Where comparison is possible between these demographics and their counterparts in WebMD and DailyStrength, we saw that Google+ users shared experiences less frequently (*P*<.001 for all such comparisons).

Educational material was shared less frequently by users aged between 35 and 44 years, 14.9% (46/308) than by users of any other age group. In particular, they shared educational material much less frequently than both the previous age group, 18 to 34 years, 25.5% (141/552), *P*<.001; and the following age group, 45 to 64 years, 34.3% (171/499), *P*<.001. Asian Google+ users, 35.75% (1010/2825), substantially shared more educational material than users of any other ethnicity (*P*=.002 vs black users, *P*<.001 vs Hispanic users, and *P*<.001 vs white users).

**Table 18:** Google+ category frequency by gender, age, ethnicity, and location.

Attribute and demographic		Total number of participants	Share experiences, n (%)	Educational material, n (%)
**Gender**				
	Male	61,479	15,234 (24.78)	16,200 (26.35)
	Female	32,082	9803 (30.56)	8029 (25.03)
**Age group (years)**				
	0-17	42	19 (45.24)	8 (19.05)
	18-34	552	189 (34.24)	141 (25.54)
	35-44	308	101 (32.79)	46 (14.94)
	45-64	499	62 (12.42)	171 (34.27)
	≥65	45	9 (20.00)	13 (28.89)
**Ethnicity**				
	Asian	2825	730 (25.84)	1010 (35.75)
	Black	72	28 (38.89)	13 (18.06)
	Hispanic	3389	1137 (33.55)	707 (20.86)
	White	17,230	5076 (29.46)	3340 (19.38)
**Region**				
	Northeast	4510	1097 (24.32)	957 (21.22)
	Midwest	4210	1310 (31.12)	716 (17.01)
	South	9532	2636 (27.65)	1913 (20.07)
	West	7959	2279 (28.63)	1708 (21.46)

## Discussion

### Principal Findings

Our analysis shows several interesting results. From our initial samples, we found that health-related posts from general social networks often shared news and educational material, and posts on health-related online forums frequently shared experiences, asked for medical advice, and requested or gave psychological support (Table 5). Our evaluation of three classification algorithms on the post content categories described by our study showed that, in terms of balanced accuracy, SVM tended to perform well on WebMD, whereas CNN performed better on DailyStrength data. Of the 2 Twitter categories used in our experiments, share experiences and share news, SVM performed the best in share experiences and CNN was the best in share news. None of the classifiers we evaluated performed particularly well when trained with the Google+ data; only the CNN classifier was able to meet our performance threshold in the Google+ educational material category. However, in the share experiences category, classifiers trained on the DailyStrength and Twitter data were able to meet our performance threshold in the Google+ share experiences category, suggesting that at least some transferability is possible with classifiers trained on other datasets.

A further analysis of our health-related online forum data showed distinct differences between users of WebMD and DailyStrength. On WebMD, we found that the majority of posts made by male users and almost half of all posts made by female users asked for advice. This would seem to contradict an earlier study that found that women were the predominant users of the internet for health advice [39], but when considering the overall number of posts from male and female WebMD users included in our study (41,422 posts by men vs 93,293 by women), we saw that posts asking for advice were still more likely to be written by a woman than a man. DailyStrength users shared experiences frequently in all demographics analyzed in our study, even more so than WebMD users; however, asking for advice was less common than on WebMD. These differences may be explained by the differences in the 2 health-related online forums; although DailyStrength offers support groups for a variety of topics, WebMD communities are often frequented by experts who can provide advice to users.

An analysis of health-related posts on general social networks, Twitter and Google+, suggested differences that they have from health-related online forums. Compared with WebMD and DailyStrength, sharing experiences, which identifies posts in which a user shared a personal experience related to a health-related topic, is far less frequent in posts from Twitter and Google+ that contain one or more of the health-related keywords used in this study. The relatively low frequency of sharing experiences in our sample of several health-related topics on general social networks compared with the frequency of sharing experiences on health-related online forums may be due to a variety of factors, such as Twitter’s lack of health-related communities because of its structure as well as WebMD’s and DailyStrength’s focus on answering medical questions and providing support, respectively. Some subsets of health-related tweets studied in other work have low proportions of sharing experiences similar to our observations, such as tweets about depression [7], schizophrenia [7], and dementia [28], as well as tweets from health-related Twitter users [8]. However, other work has shown that the proportion can be much higher, such as in tweets about dental pain [40] and prescription drug use [29]. Many health-related topics had high proportions of posts that shared experiences in our Google+ data, for example, *headache*, 93.22% (6572/7050); *migraine*, 78.77% (2029/2576); *insomnia*, 71.41% (2430/3403); *cold sore*, 58.0% (370/638); and *diazepam*, 51.1% (95/186). This suggests that the proportion of sharing experiences in health-related posts may be highly dependent on the topic or topics studied; thus, our findings on the share experiences category may not generalize to other studies on health-related social media posts.

Our comparison of results between our stratified sample of Twitter data with tweets from suspected bots removed and our full Twitter dataset showed that automated accounts had a significant impact on the share news category. Other work has also shown that bots can have an effect on health-related Twitter conversations, particularly on the subject of vaccination. Bots post both pro- and antivaccine tweets [41] and retweet vaccine-related tweets at higher frequencies than human users [42]. The use of bots in this manner amplifies the debate and further polarizes the communities involved. It is clear that bot activity must be considered when analyzing health-related conversations on Twitter.

The differences in how often educational material is shared on Google+ between the demographics we studied highlight potential targets for informational health care campaigns. A health care campaign is a health care–related broad nationally or subnationally driven, led, or coordinated activity [43]. Users in the age demographic of 35 to 44 years, who share educational material less often than other age groups, may benefit from being provided with medical information that they are not aware of. Demographics that share educational material more frequently than others, such as Asian Google+ users, may also be of interest to medical experts. If a further analysis of the educational material shared by these groups shows that the information is inaccurate or misleading, providing correct information may benefit them.

Our results provide useful information that can help health care providers to reach the right demographic group. For example, researchers looking for clinical trial participants can use health-related online forums, where many posts are about sharing experiences. Moreover, demographic-specific results can help guide the targeted educational campaigns. As an example, male WebMD users ask specific medical advice questions more often than females, so male WebMD users may be more receptive to a campaign offering advice from medical experts.

The classifier models used in this study can also be useful for researchers who want to study posts that contain the categories we studied. For example, a researcher who wants to study experiences about a particular drug can use these classifiers to find posts that share experiences from a larger dataset of posts that mention that drug. As another example, a researcher who wants to find out which disorders are frequently mentioned among users who share news can use a classifier to gather a dataset of news-sharing posts. In general, we provided researchers with tools that enable them to answer hypotheses and do research on the subject of health-related social media posts. These tools are provided by the description of our methodology, which describes how one might build these classifier models, and by trained classifier models that are available on request. Similar tools may also be applicable to the categories in the scheme proposed by Lopes and Da Silva [9]. We leave this as future work.

### Limitations

As users of health-related social media use an informal writing style, our selected 274 words to filter Twitter and Google+ as described in the Methods section may not cover all health-related posts or their variability in topics. For example, the abbreviation *IUI* (intrauterine insemination) is widely used in health-related posts but not included in the health-related keyword list. Another limitation is the different uses of terms used to filter Twitter and Google+. For example, the word “cancer” yields many tweets that talk about zodiac signs.

We found that some Twitter categories have a high proportion of tweets from automated accounts. Although we have attempted to filter out tweets from such accounts, some such tweets may still exist in the data used in our analysis, and tweets from legitimate accounts may have been filtered out. Our initial evaluation of bot prevalence also found that the educational material category had a high proportion of tweets from bots. This may be also true of that category in the Google+ data, which was not filtered for bots; thus, those results may not accurately represent the demographics studied.

Our demographic populations may not be fully representative of all users from the sources in our study. As shown in Table 1, some of our demographics were estimated using classifiers, and these estimates are not always correct. Other demographics in our study are optionally reported by users. This introduces a bias toward users who choose to report their age, gender, and/or location, as noted in our results from Google+. We also assumed these reported demographics are correct for each such user.

### Conclusions

In this study, we analyzed the content shared in two different types of health-related social media: health-related online forums and general social networks. For the two types of health-related social media, we manually identified 4 post categories: share experiences, ask for specific medical advice, request or give psychological support, and about family; and we additionally identified 5 categories for general social networks: share news, jokes, advertisements, personal opinion, and educational material. After labeling randomly selected data for each source, we built classifiers for each category. Finally, we made demographic-based content analyses where possible.

## Abbreviations

API: application programming interface
CNN: convolutional neural network
SVM: support vector machine
